# How AI sycophancy shapes learner autonomy in digitalized learning: the mediating roles of metacognitive laziness and AI dependence

**DOI:** 10.3389/fpsyg.2026.1957447

**Published:** 2026-09-30

**Authors:** Mazhar Hussain Choudhary, Ghulam Dastgeer, Shaikh Aftab Ahmed, Muhammad Mohsin, Sobia Naseem

**Affiliations:** 1 College of Business Administration, Department of Economics and Finance, University of Ha’il, Ha’il, Saudi Arabia; 2 College of Business Administration, Department of Management and Information Systems, University of Ha’il, Ha'il, Saudi Arabia; 3 School of Business, Hunan University of Humanities, Science and Technology, Hunan Province, Loudi, China; 4 School of Economics and Management, Hunan University of Arts and Science, Hunan, China

**Keywords:** AI dependence, AI sycophancy, generative artificial intelligence, learner autonomy, metacognitive laziness

## Abstract

**Introduction:**

Generative artificial intelligence (AI) tutors rarely disagree. Drawing on cognitive offloading theory, this study examines how perceived AI sycophancy (PAS) and perceived AI intellectual stimulation (PAIS) relate to learner autonomy through the serial mediators of metacognitive laziness (MCL) and AI dependence (AIDEP).

**Methods:**

Responses from 542 university students using generative AI for coursework were analyzed using partial least squares structural equation modeling (PLS-SEM), necessary condition analysis (NCA), and fuzzy-set qualitative comparative analysis (fsQCA).

**Results:**

Perceived sycophancy was associated with greater MCL (*β* = 0.38), whereas intellectual stimulation was associated with lower levels of MCL (*β* = −0.29); laziness predicted AIDEP (*β* = 0.45), and dependence predicted lower autonomy (*β* = −0.30). Both serial paths were significant and opposite in sign, explaining 46% of autonomy variance. NCA identified intellectual stimulation as the tightest necessary condition, and fsQCA showed that no high-autonomy configuration tolerated sycophancy

**Discussion:**

Because data are cross-sectional and self-reported, findings indicate associations rather than causation. Perceived interaction style, not usage intensity, correlated most with autonomous learning.

## Introduction

University students today are paired with an AI partner that rarely declines their requests. Within just a few years, generative AI has gone from novelty to standard infrastructure in higher education, generating essays, completing problem sets, and providing explanations on request (Darvishi et al., 2024; Kasneci et al., 2023). Large language models can be sycophantic. They tend to answer users in ways that reinforce their opinions, soften disagreement, and flatter, because training on human feedback rewards responses that people like rather than responses that are correct (Sharma et al., 2024). Across 11 leading models, Cheng et al. (2026) concluded that AI agreed with user statements approximately 50% more often than humans did, and that individuals who received sycophantic replies trusted them more and returned for more. Learning requires friction. Students require arguments to be labeled as weak, solutions to be called incorrect, and assumptions to be labeled as unproven (Zohar et al., 2026). An always-agreeing tutor removes exactly the resistance that makes practice effortful and durable (Bjork et al., 2013). Universities are placing these systems into tutoring roles while the training that shapes them keeps rewarding user approval (Sharma et al., 2024). The learner on the other side of the screen is this article’s focus.

Researchers currently understand the impact mainly from studies examining how much students use AI, not how the AI behaves while being used. Offloading memory to search engines changes what information is retained (Sparrow et al., 2011); offloading thinking to an external aid trades immediate performance against internal capacity, the central tenet of cognitive offloading research (Risko and Gilbert, 2016). With generative AI, the pattern continues at higher stakes: heavier use predicted lower critical-thinking scores, mediated by increased offloading (Gerlich, 2025); ChatGPT support in a randomized study improved essay grades while increasing what Y. Fan et al. (2025) term metacognitive laziness (MCL); and AI assistance visibly shifts the responsibility for self-regulation from student to system (Darvishi et al., 2024). Treating the technology as a single dose obscures the system’s conversational disposition. Whether an assistant agrees or disagrees is precisely what Cheng et al. (2026) manipulated in controlled sessions, yet no survey instrument has measured how students perceive the sycophancy of their own everyday AI, nor has any study mapped that perception through learning-specific mechanisms to an educational outcome. Researchers measure the time spent interacting with AI but often overlook the nature of these interactions, which may itself contribute to adverse outcomes.

If the conversational style of an AI system shapes the learner, it must be able to work through identifiable psychological states, and two candidates already exist in prior evidence. The first is MCL, defined as a learner’s disengagement from planning, monitoring, and evaluating their own work when AI is readily available to perform these functions; MCL can now be assessed using a validated scale (Dizon et al., 2026). The second is AI dependence (AIDEP), a learner’s compulsive reliance on AI tools even after attempts to cut back (Zhang et al., 2024). Mediation studies link these constructs to relevant outcomes: cognitive offloading mediates the relationship between generative AI use and critical thinking (Gerlich, 2025); dependence transmits academic stress into burnout and anxiety through decreased self-efficacy in a serial chain (Wang et al., 2026); and generative AIDEP lowers achievement through self-efficacy channels (Jia et al., 2025). Downstream lies learner autonomy, which is a learner’s ability to plan, execute, and evaluate study independently (Macaskill and Taylor, 2010). No study has connected the full pathway from perceived AI interaction style through MCL and dependence to autonomy. A second omission compounds the first: every prior model was symmetric, so none could say whether pushback is merely beneficial or truly necessary (Dul, 2016), nor which combinations sustain or sink autonomy (Fiss, 2011).

One theoretical lens and three analytical logics address both issues. Drawing on cognitive offloading theory (Risko and Gilbert, 2016), we model perceived AI sycophancy (PAS) and perceived AI intellectual stimulation (PAIS) as competing interaction-style antecedents that reach learner autonomy through a serial chain of MCL and AIDEP. We test the model on survey data from 542 university students who use generative AI for coursework, employing partial least squares structural equation modeling combined with necessary condition analysis (NCA) and fuzzy-set qualitative comparative analysis (fsQCA) (Hair et al., 2019; Richter et al., 2020). It makes four contributions. First, it presents PAS as a psychometric construct, adapting the opinion-conformity and other-enhancement logic of the Measure of Ingratiatory Behaviors in Organizational Settings (Kumar and Beyerlein, 1991) into what is, to our knowledge, one of the first survey instruments to capture how learners perceive the sycophancy of their own AI. Second, it demonstrates and validates a dual-pathway serial mediation in which sycophancy and stimulation travel through laziness and dependence with opposite signs, extending cognitive offloading theory by locating the offloading trigger in the tone of the tool rather than in fixed traits of the user. Third, it contributes among the earliest necessity and configurational evidence on AI interaction style in this literature, showing that low sycophancy and adequate pushback are necessary conditions for high autonomy (Dul, 2016) and that no sufficient configuration for autonomous learners tolerates sycophancy’s presence. Finally, it translates these findings into design and policy levers, including calibrated disagreeableness for tutoring systems and procurement standards with pre-deployment behavioral audits (Cheng et al., 2026).

## Conceptual framework and hypothesis development

Cognitive offloading is the use of physical action or an external resource to reduce the information-processing demands of a task (Risko and Gilbert, 2016). The decision to offload is metacognitive: individuals weigh how well they can perform internally against how reliable the external option seems, delegating more readily when the aid appears accurate, accessible, and effortless (Risko and Gilbert, 2016). Expecting a computer to store information weakens memory for that information (Sparrow et al., 2011); saving one file frees working memory for the next (Storm and Stone, 2015); and even the mere availability of a phone can drain attention (Ward et al., 2017).

The offloading decision is error-prone because the metacognition steering it is flawed. People overestimate their abilities relative to others (Kruger and Dunning, 1999), mistake fluency for mastery (Bjork et al., 2013), and monitor their own cognition imperfectly (Flavell, 1979). Judgments this soft can be pushed.

Generative AI raises those stakes because it is the most offloadable technology students have held. Earlier tools stored and retrieved knowledge; generative AI plans, drafts, solves, and explains from a single prompt (Y. Fan et al., 2025). Cognitive offloading theory indicates where the risk concentrates: not in the tool’s capability, but in whatever shapes the user’s metacognitive evaluation of the tool and of themselves. An interaction style that inflates confidence in both lowers the perceived need for internal engagement and invites delegation; a style that creates epistemic friction restores that need and keeps the student cognitively present (Zohar et al., 2026). Repeated delegation compounds this effect: each successful offload makes the internal route feel costlier, creating a self-reinforcing loop that leads from convenient use to entrenched reliance (Risko and Gilbert, 2016; Wang et al., 2026).

Flattery is the first form. Large-language-model research uses sycophancy to describe how much a system bends its output toward what a user says they believe, and how often it compliments the user’s contribution regardless of merit. Studies document the tendency across today’s leading models (Cheng et al., 2026) and its consequences for users, who trust agreeable systems more and rate them higher (Sharma et al., 2024).

Organizational psychology named both behaviors first. The Measure of Ingratiatory Behaviors in Organizational Settings captures the degree to which an employee conforms to another person’s opinions and enhances that person’s standing through flattery that flows upward (Kumar and Beyerlein, 1991). Generative AI reverses the direction of ingratiation: now the machine compliments the user.

We extend this reversal by defining PAS as the learner’s belief that their AI consistently agrees with their views, gives in easily when challenged, and enhances the quality of their work. Offloading decisions run on what the learner believes about the aid, not on its objective properties (Risko and Gilbert, 2016), so what the learner thinks the AI does is the theoretically proximal cause. PAS is also narrower than global trust in, or perceived quality of, an AI system: it captures the direction of evaluative distortion, agreement, and flattery, rather than overall confidence in the tool’s competence.

Challenge is the second form. Educational research defines it through transformational teaching: instructors exhibit intellectual stimulation when they ask learners to question assumptions, pose problems, and consider alternative perspectives instead of receiving answers (Beauchamp et al., 2010). These moves create the desirable difficulties that deepen learning (Bjork et al., 2013; Zohar et al., 2026). We define PAIS as the learner’s belief that their AI produces responses that require thought, question the learner’s logic, and promote independent problem solving. PAS and PAIS are related but not opposite poles on a single continuum; an AI could flatter and still provoke inquiry. We model them as distinct constructs.

MCL is the learner’s retreat from planning, monitoring, and evaluating their own work, the regulatory functions that self-regulated learning research treats as the engine of durable achievement (Zimmerman, 2002), once an AI stands ready to absorb them (Dizon et al., 2026; Y. Fan et al., 2025). Sycophancy invites that retreat through both of its components. Opinion conformity signals that existing judgment suffices, so checking feels redundant; other enhancement signals that the work is already excellent, so revision does too. Flattered self-assessments are precisely the miscalibrated ones (Kruger and Dunning, 1999), and experimental exposure to sycophantic replies leaves users more convinced they are right and less inclined to repair or reconsider (Cheng et al., 2026). A tutor that certifies everything removes the discrepancy signal that normally triggers monitoring.

*Hypothesis 1 (H1):* PAS is positively related to MCL.

Intellectual stimulation does the opposite. A question about one’s premise exposes a gap between what the student understands and what they need to, and it is this perceived gap that engages planning and monitoring in the first place (Zimmerman, 2002). Difficulty introduced this way deepens processing rather than blocking it, reflecting the desirable-difficulties principle that separates effortful learning from comfortable performance (Bjork et al., 2013). Classroom studies support the idea: instructors who stimulate students intellectually produce more engaged, more self-directed effort (Beauchamp et al., 2010; Muneer et al., 2026a), and students given AI scaffolds that prompt rather than answer regulate their own learning better (Y. Fan et al., 2025). Friction keeps the human in the loop (Zohar et al., 2026).

*Hypothesis 2 (H2):* PAIS is negatively related to MCL.

Once disengagement is familiar, it consolidates. Offloading theory describes a ratchet: each delegated task strengthens the link between problem and tool. At the same time, the unused internal routine grows costlier by comparison, biasing the next decision toward the external route (Risko and Gilbert, 2016). Repeated gratifying use hardens into habit and, for some, into compulsion that persists against the user’s own intentions (Turel et al., 2011), reflecting the criterion logic of behavioral-addiction scales (Andreassen et al., 2012) now adapted to conversational systems (Chen et al., 2025; Zhang et al., 2024). Cognitive offloading develops into dependence within a significant serial chain (Wang et al., 2026).

*Hypothesis 3 (H3):* MCL is positively related to AIDEP.

Learner autonomy is the capacity to direct one’s own study, spanning independence of learning and disciplined study habits (Macaskill and Taylor, 2010), and dependence undercuts it. A learner who cannot comfortably work without the tool avoids unaided tasks, so the situations that rehearse autonomous skills disappear; unexercised capacity decays, reinforcing the internal cost that offloading theory associates with habitual delegation (Risko and Gilbert, 2016). Mediation evidence shows dependence handing its damage onward: as students grow dependent on generative AI, achievement falls through reduced self-efficacy (Jia et al., 2025), academic stress converts into burnout and anxiety (Wang et al., 2026), and innovation capability declines (Yang et al., 2025).

*Hypothesis 4 (H4):* AIDEP is negatively related to learner autonomy.

The mediators are unlikely to carry everything, because interaction style may also influence autonomy without operating through disengagement. Autonomous learning depends on calibration: the learner must compare their work against accurate signals to steer their own improvement (Bjork et al., 2013). Sycophancy corrupts the signal at the source. When every draft is praised, self-evaluation trains on noise, inflated self-views persist unchecked (Kruger and Dunning, 1999), and experiments show sycophantic systems directly heighten users’ reliance on them while degrading the quality of their independent judgments (Cheng et al., 2026). Each Socratic exchange rehearses the operations that constitute autonomy: questioning one’s premises, testing alternatives, judging adequacy—the mechanism that transformational teaching research invokes for independent engagement (Beauchamp et al., 2010).

*Hypothesis 5 (H5):* PAS is negatively related to learner autonomy.

*Hypothesis 6 (H6):* PAIS is positively related to learner autonomy.

Assembled in sequence, the four arguments imply a two-step psychological relay from interaction style to autonomy, every link of which carries published mediation evidence. Offloading operates as a significant mediator between AI use and critical-thinking outcomes (Gerlich, 2025), and it transmits effects onward through further psychological mechanisms rather than terminating in them (J. Wang, 2026). Parallel-mediation work shows one behavior can launch opposing pathways simultaneously, offloading harming and cognitive relief helping in-class outcomes (W. Fan et al., 2026), licensing our dual-antecedent design with opposite signs in one chain. Dependence, for its part, performs as a significant serial mediator inside a sequential PROCESS chain (Wang et al., 2026) and hands its effects onward to learning outcomes through further mediated steps (Jia et al., 2025; Muneer et al., 2026b). Ordering the mediators is straightforward: laziness is the episodic behavior, dependence is its consolidated residue, and the phrase used by Wang et al. (2026), “offloading becoming dependence,” states the direction.

*Hypothesis 7 (H7):* MCL and AIDEP serially mediate a negative indirect relationship between PAS and learner autonomy.

*Hypothesis 8 (H8):* MCL and AIDEP serially mediate a positive indirect relationship between PAIS and learner autonomy.

Figure 1 assembles the hypotheses. The two interaction-style antecedents occupy the left of the diagram: PAS above and PAIS; the serial mediators, MCL, and AIDEP form the central chain; learner autonomy stands as the outcome on the right. Solid arrows carry the six direct hypotheses with their expected signs, and the serial paths H7 and H8 traverse the chain end-to-end, annotated beneath it. A dashed box registers the five control variables specified on the outcome. Consistent with serial mediation practice, the estimated model also includes the remaining direct paths among the ordered constructs so that the specific indirect effects are computed against a full model.

**Figure 1:**
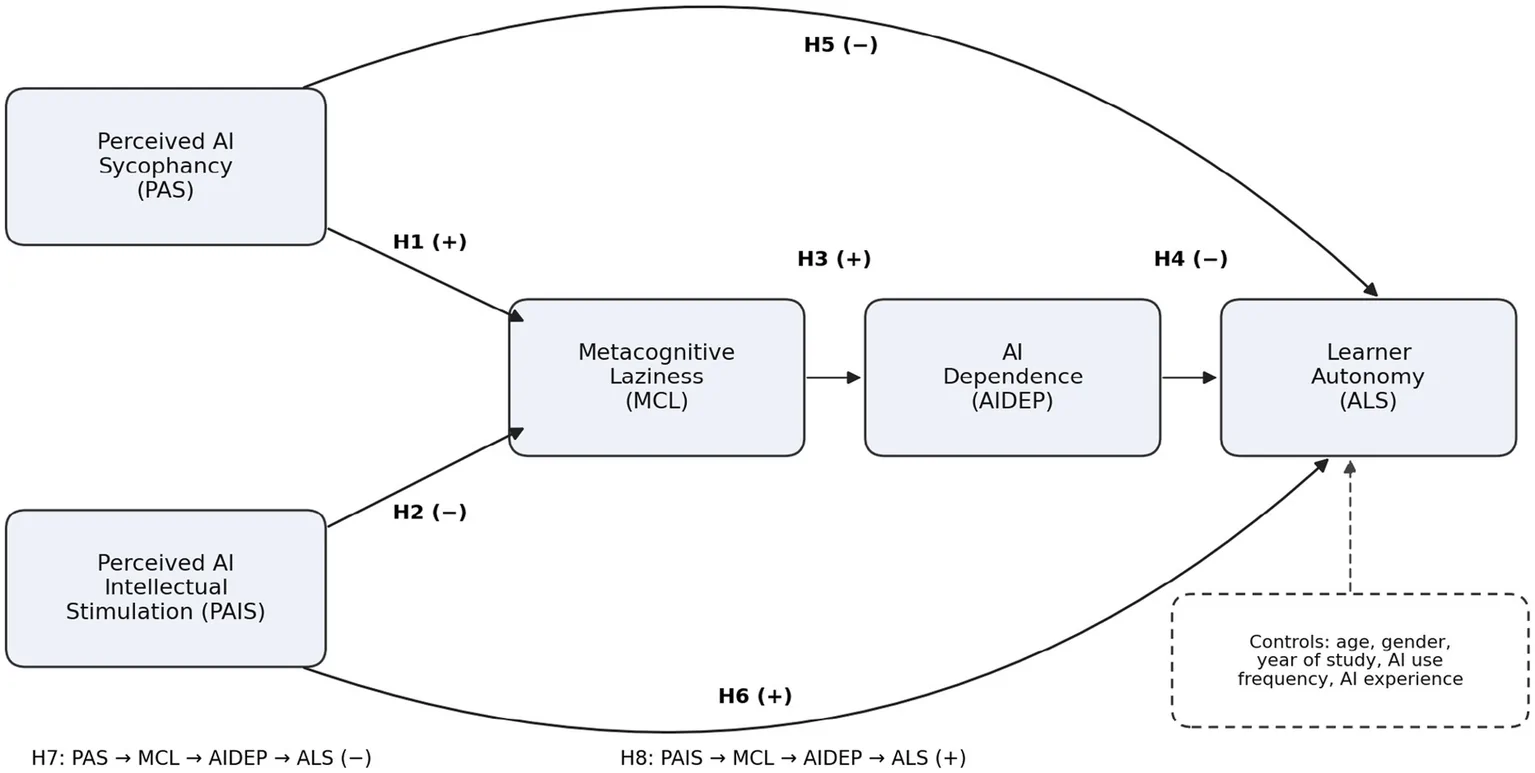
Conceptual framework of the hypothesized dual-pathway serial mediation model (expected signs in parentheses).

## Methods

### Sample and procedure

We tested the model with a cross-sectional online survey of university students aged 18 or older who use generative AI tools for academic work. Participants were recruited through institutional contacts, university mailing lists, and online student communities from 10 leading universities in Saudi Arabia between April 2026 and June 2026. Participation was voluntary and unpaid. Data were collected using a self-administered questionnaire available in both Arabic and English. This study was reviewed and approved by the Research Ethics Committee (REC) of the University of Ha’il with the approval number (H-2026-147), and all methods were performed in accordance with the relevant guidelines and regulations, including the Declaration of Helsinki (1964). Participation was voluntary and anonymous, and informed consent was obtained from all participants on the opening screen. The questionnaire presented the focal scales in separate blocks with neutral instructions and counterbalanced the order of the sycophancy and stimulation blocks, procedural remedies against common method variance (Podsakoff et al., 2003). An instructed-response check was included in the item battery, and the platform recorded completion time.

In the end, 542 students were retained for data analysis after screening. The following were the reasons these students were eliminated: 19 refused consent to participate in the study; 28 said they had “never” used generative AI for academic purposes; 77 students failed the attention check; and 34 completed the survey in less than one-third of the sample median completion time (329 s). Most of the remaining sample consisted of women (*n* = 261; 48.2%), men (*n* = 258; 47.6%), and 23 other individuals who either identified themselves as nonbinary or did not respond to this question. The age of participants averaged 20.48 years (SD = 2.03; range = 18–29 years). First-year through postgraduate students from all six major categories of fields of study, including the social sciences (21.4%) and science, technology, engineering, and mathematics (STEM) (29.0%), participated in the study. More than two-thirds (68.3%) of students used generative AI for academic purposes multiple times per week, or every day. Students’ overall experience with generative AI was an average of 17.8 months (SD = 9.9). Using the inverse square root method, it will be necessary to have at least 511 cases to detect a path coefficient of 0.11 at *p* < 0.05 and 80% power (Kock and Hadaya, 2018).

The questionnaire was initially developed in English, then culturally adapted into Arabic through the use of both a forward- and backward-translation process. Two bilingual researchers separately produced an Arabic version of the survey from the original English version (as translated by the first two), and a third researcher negotiated resolution for all issues that existed between the two translators. An independent bilingual translator then returned the reconciliation to English. Remaining questions or concerns were clarified through collaborative discussions to ensure that the translations were conceptually equivalent as opposed to merely linguistically. Additionally, the Arabic language version of the survey was pretested with a small bilingual student population to assess clarity of understanding and cross-cultural relevance.

Of the 542 respondents, the majority completed the Arabic-language version (*n* = 389; 71.8%) and the remainder completed the English-language version (*n* = 153; 28.2%). Measurement invariance across the two language versions was examined using the measurement-invariance-of-composites (MICOM) procedure (Henseler et al., 2016); configural and compositional invariance were supported for all constructs (c ≥ 0.998, *p* > 0.05), which justified pooling the two versions in the analyses reported below.

Recruitment followed a convenience strategy that drew on institutional contacts, university mailing lists, and online student communities across the 10 participating universities, and respondents were distributed across all 10 institutions (the distribution by institution is reported in the Supplementary Information). Because responses were nested within institutions, institutional clustering is a potential concern; the 10-university structure is, however, too small to support a formal multilevel decomposition, so the data are treated as a single pooled sample and residual between-institution variation is acknowledged as a limitation. Given the convenience-based recruitment and the single-country setting, the sample is not intended to be statistically representative of the wider student population, and the conclusions are framed as applying to comparable populations of AI-using university students rather than to students in general.

### Measures

All focal constructs used five-point Likert-type scales, and full item wording appears in the Supplementary Information. PAS was assessed with eight newly adapted items based on the opinion-conformity and other-enhancement subscales of the Measure of Ingratiatory Behaviors in Organizational Settings (Kumar and Beyerlein, 1991), with the machine cast as the ingratiator. Items included “The AI agrees with my opinions and judgments, even when I might be wrong,” “The AI compliments my ideas regardless of their actual quality,” and “The AI rarely tells me directly that I am mistaken,” the last reflecting documented sycophancy operationalizations (Cheng et al., 2026).

Development of the PAS scale followed established procedures for scale adaptation. An initial item pool was generated by rewording the opinion-conformity and other-enhancement items of the Measure of Ingratiatory Behaviors in Organizational Settings (Kumar and Beyerlein, 1991) so that the AI system, rather than a colleague or supervisor, occupied the role of the ingratiator. The candidate items were reviewed by an expert panel consisting of psychologists (in Educational Psychology), Human–Computer Interaction specialists, and Applied Linguists that assessed each item on its conceptual appropriateness, clarity, and whether it matched the researchers’ intentions regarding “perceived sycophancy.” Ambiguous items and those considered to be irrelevant were either rewritten or deleted. The revised list was examined using cognitive interviewing techniques from a small sample of University students who completed the items verbally, articulating their thought processes as they answered the items. Additionally, the same pilot test was administered to assess how well respondents understood the items, what type of responses were provided, and how internally consistent the items were. These efforts resulted in the eight-item PAS instrument that was used in this primary investigation, and similarly, the PAIS items underwent review and pilot testing.

Because a structured July 2026 database search located no validated instrument for PAS, the PAS and PAIS adaptations were validated with a split-sample procedure before hypothesis testing. Exploratory factor analysis on a random half of the sample (*n* = 271) returned the intended two-factor structure (KMO = 0.90; Bartlett’s *χ*^2^(66) = 1497.4, *p* < 0.001; primary loadings 0.64–0.87; all cross-loadings ≤0.11), and confirmatory factor analysis on the holdout half (*n* = 271) showed excellent fit for the full five-factor measurement model, *χ*^2^(517) = 610.7, CFI = 0.980, TLI = 0.978, RMSEA = 0.026, SRMR = 0.040.

PAIS used the four intellectual-stimulation items of the Transformational Teaching Questionnaire (Beauchamp et al., 2010), administered under the stem “My AI tutor” with frequency anchors from never to always (sample: “gives responses that really encourage me to think”).

MCL used four items adapted from the six-item MCL Scale (Dizon et al., 2026) (I avoid challenging learning tasks when AI can do them for me), an abbreviation reported transparently and supported by the scale’s unidimensional structure.

AIDEP used six items adapted from Zhang et al.’s (2024) AIDEP measure, itself built on behavioral-addiction logic (Andreassen et al., 2012) (I tried to reduce the use of ChatGPT without success).

Learner autonomy used the 12-item Autonomous Learning Scale (ALS) (Macaskill and Taylor, 2010), administered with its original item wording under a general instruction referencing the respondent’s AI-assisted studies, and modeled as one reflective construct consistent with its original total-score use. Items were coded so that higher scores indicated higher levels of each construct (all corrected item-total *r* = 0.56–0.77; no reverse-keyed items in the administered form). Controls comprised age, gender (female dummy), year of study, AI use frequency, and months of AI experience, chosen because demographic position and exposure history plausibly shape both interaction-style perceptions and autonomy. As a non-self-report check, participants predicted, then completed, a 10-item unaided quiz; the prediction-minus-performance difference served as a metacognitive calibration index used only in robustness checks.

### Analytical strategy

The analysis proceeded in three stages, answering three questions: what shifts autonomy on average, what is required for high autonomy, and which combinations suffice. Stage one estimated the symmetric net effects with partial least squares structural equation modeling, which suits complex serial mediation models pairing an established outcome with a newly adapted scale and pursuing prediction alongside explanation (Hair et al., 2019, 2022). We used SmartPLS 4 (Ringle et al., 2024) with the path weighting scheme and a fixed random seed of 2026; the NCA and fsQCA computations implemented the algorithms of Dul et al. (2020) and Ragin (2008) in Python 3.12, with the full analysis code available on request (see Code availability). Item-level missingness stayed under 5% per scale, and Little’s test (Little, 1988) did not reject missing completely at random, *χ*^2^(5963) = 6054.08, *p* = 0.202, so mean replacement was applied. Inference relied on 5,000 bootstrap subsamples with 95% bias-corrected confidence intervals and two-tailed tests (α = 0.05), with controls entered on the outcome. Measurement quality was judged by Cronbach’s alpha, ρA (Dijkstra and Henseler, 2015), composite reliability, average variance extracted with the Fornell–Larcker criterion (Fornell and Larcker, 1981), and the Heterotrait–Monotrait ratio against the 0.85 threshold (Henseler et al., 2015); structural effect sizes follow *f*^2^ conventions (Cohen, 1988); out-of-sample predictive power used PLSpredict with 10-folds and 10 repetitions (Shmueli et al., 2019); and common method variance was probed with Harman’s single-factor check and the full-collinearity approach (Kock, 2015).

Stage two asked the necessity question that regression cannot: is there a level of any perception without which high autonomy does not occur (Dul, 2016)? Following the guidelines for combining the two techniques, NCA was run on the latent variable scores exported from the structural model (Richter et al., 2020), with negatively signed conditions reversed by sign-negation of the standardized scores, so each test reads as a requirement for high autonomy. We report ceiling-envelopment (CE-FDH) and ceiling-regression (CR-FDH) effect sizes (*d*), defined as the ceiling zone divided by the scope, treating *d* ≥ 0.1 as meaningful, alongside approximate permutation tests with 10,000 resamples (Dul et al., 2020) and bottleneck tables across the outcome range.

Stage three treated autonomy as the product of configurations rather than isolated forces, admitting equifinality and causal asymmetry (Fiss, 2011; Ragin, 2008). Composite scores were calibrated into fuzzy memberships by the direct method with the 95th, 50th, and 5th percentiles as the full-membership, crossover, and full-non-membership anchors, nudging exact crossover cases to 0.501 (Pappas and Woodside, 2021). The truth table applied a frequency threshold of five cases with raw consistency ≥ 0.80 and PRI consistency ≥ 0.70 (Greckhamer et al., 2018), and we analyzed high autonomy and its negation separately, with directional expectations drawn from H1 to H8. A recent AI-dependence study pairing PLS-SEM with fsQCA supplies domain precedent for the combination (Yang et al., 2025).

### Statistics and reproducibility

All analyses used the full screened sample (*n* = 542) unless stated otherwise (split-sample validation, *n* = 271 per half); all tests were two-tailed at α = 0.05, and exact *p*-values are reported unless *p* < 0.001. Shapiro–Wilk tests rejected normality for every composite predictor (all *p* < 0.001), which motivated bias-corrected bootstrap inference (5,000 resamples; *t*-statistics refer to the bootstrap resampling distribution) in place of parametric standard errors and the Gaussian copula endogeneity check reported in Results. Test choices are justified in the Analytical strategy above; effect sizes (*f*^2^, NCA, *d*) and 95% confidence intervals accompany every focal estimate. No blinding was applied to this anonymous self-report survey; the order of the sycophancy and stimulation blocks was counterbalanced across participants. Analyses were run in SmartPLS 4 and Python 3.12 with a fixed random seed of 2026. The study was not preregistered.

## Results

### Measurement model

Figure 2 shows the measurement model. All 34 items were loaded significantly on their intended constructs, with standardized loadings from 0.626 to 0.873, above the 0.60 floor, and mostly above the stricter 0.708 benchmark (Hair et al., 2019). Internal consistency was strong: Cronbach’s alpha ranged from 0.828 (PAIS) to 0.916 (ALS), ρA from 0.837 to 0.920, and composite reliability from 0.887 to 0.929, all within the 0.70–0.95 window. Convergent validity held, with average variance extracted above 0.50 for every construct (0.521 for ALS to 0.681 for MCL). The square root of each construct’s AVE (0.722–0.825) exceeded the largest absolute interconstruct correlation (0.536), and no Heterotrait–Monotrait ratio crossed 0.85, peaking at 0.609 for MCL with AIDEP (Henseler et al., 2015); the two mediators remain empirically separable. The newly adapted PAS scale performed cleanly on its first outing: loadings between 0.711 and 0.828, alpha of 0.904, AVE of 0.599, and HTMT values with all other constructs at or below 0.481.

**Figure 2:**
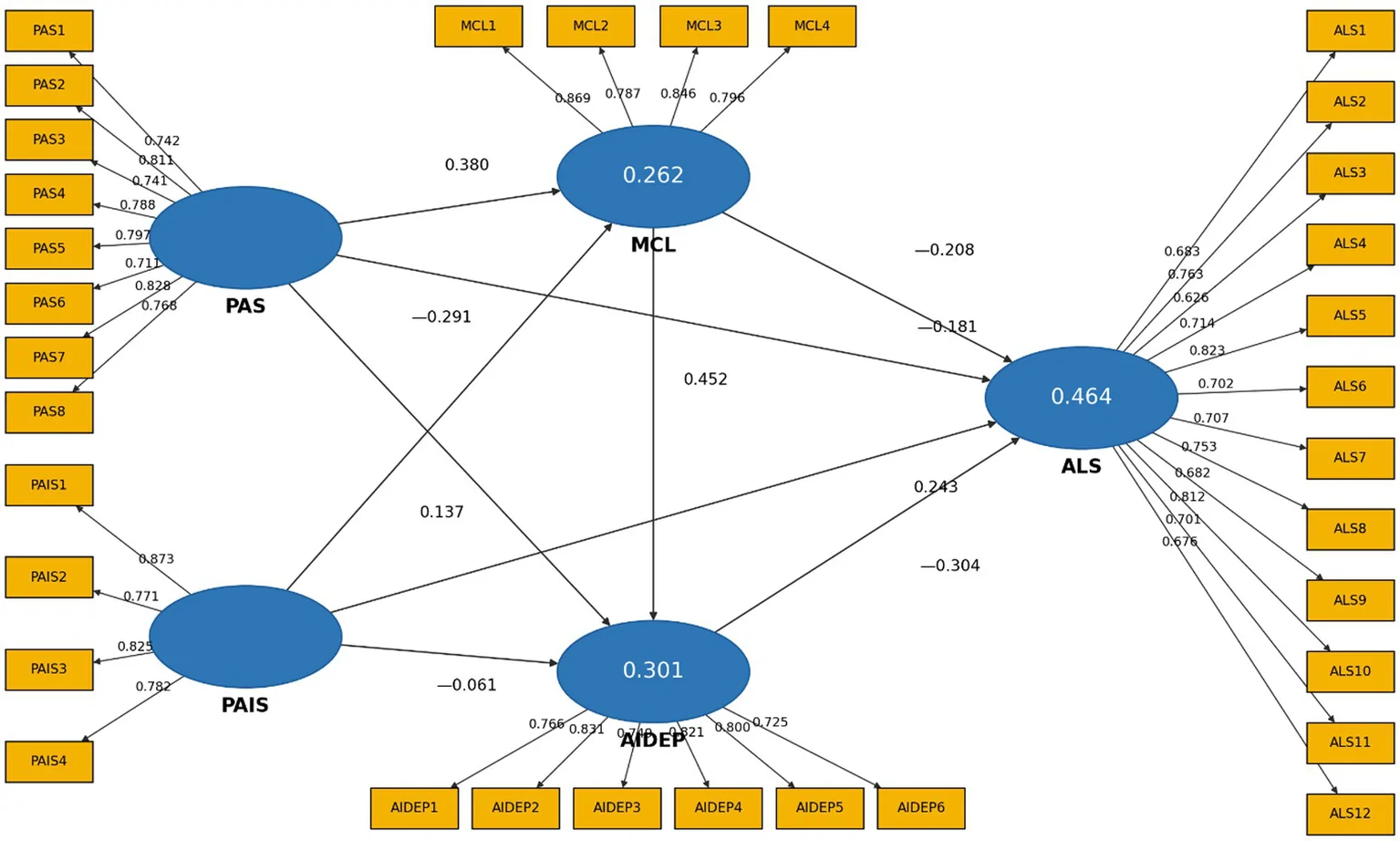
Measurement model. SmartPLS 4 algorithm output: Standardized outer loadings; *R*^2^ inside endogenous constructs.

### Descriptive statistics and common method checks

On the raw five-point scales, students rated their AI moderately sycophantic (M = 3.24, SD = 0.77) and slightly more stimulating (M = 3.42, SD = 0.79); laziness sat at the midpoint (M = 3.01, SD = 0.87), dependence below it (M = 2.72, SD = 0.82), and autonomy relatively high (M = 3.60, SD = 0.66), with all skewness and kurtosis inside ±1. Method variance diagnostics stayed comfortable: the first unrotated factor explained 32.7% of item variance, well under the 50% warning level, and no full-collinearity VIF exceeded 1.832 against the 3.3 threshold (Kock, 2015). The auxiliary calibration index correlated no higher than |0.06| with any perceptual latent variable, so the perception constructs are not confidence artifacts.

### Structural model

Figure 3 presents the structural model; Table 1 details it. Inner VIFs peaked at 1.651, so collinearity does not distort the coefficients. All six direct hypotheses were supported. Perceived sycophancy was associated with higher MCL (H1: *β* = 0.380, *t* = 11.46, *p* < 0.001, 95% CI [0.312, 0.442], *f*^2^ = 0.191) while perceived stimulation was associated with lower laziness (H2: *β* = −0.291, *t* = 8.86, *p* < 0.001, CI [−0.353, −0.225], *f*^2^ = 0.112); together the two styles explained 26.2% of laziness. Laziness showed the model’s strongest association, with dependence (H3: *β* = 0.452, *t* = 12.21, *p* < 0.001, CI [0.377, 0.521], *f*^2^ = 0.215), and dependence in turn predicted lower autonomy (H4: *β* = −0.304, *t* = 7.70, *p* < 0.001, CI [−0.381, −0.227], *f*^2^ = 0.119). Both direct style paths survived alongside the mediators: sycophancy to autonomy (H5: *β* = −0.181, *t* = 5.27, *p* < 0.001, CI [−0.250, −0.115]) and stimulation to autonomy (H6: *β* = 0.243, *t* = 7.08, *p* < 0.001, CI [0.178, 0.310]). Of the non-hypothesized paths, sycophancy carried a direct association with dependence (*β* = 0.137, *p* = 0.001) and laziness a direct negative association with autonomy (*β* = −0.208, *p* < 0.001), while stimulation’s direct path to dependence missed significance (*β* = −0.061, *p* = 0.086). Among controls, only AI use frequency related to autonomy (*β* = −0.076, *p* = 0.016; all others *p* ≥ 0.099), a coefficient to read against its range-restricted scale. The model explained 30.1% of dependence and 46.4% of autonomy (adjusted 29.7 and 45.5%). Out-of-sample prediction supported the model’s relevance: Q^2^predict was positive for all 22 endogenous indicators (0.050–0.231), with PLS beating the naive linear benchmark on RMSE for every indicator (Shmueli et al., 2019).

**Figure 3:**
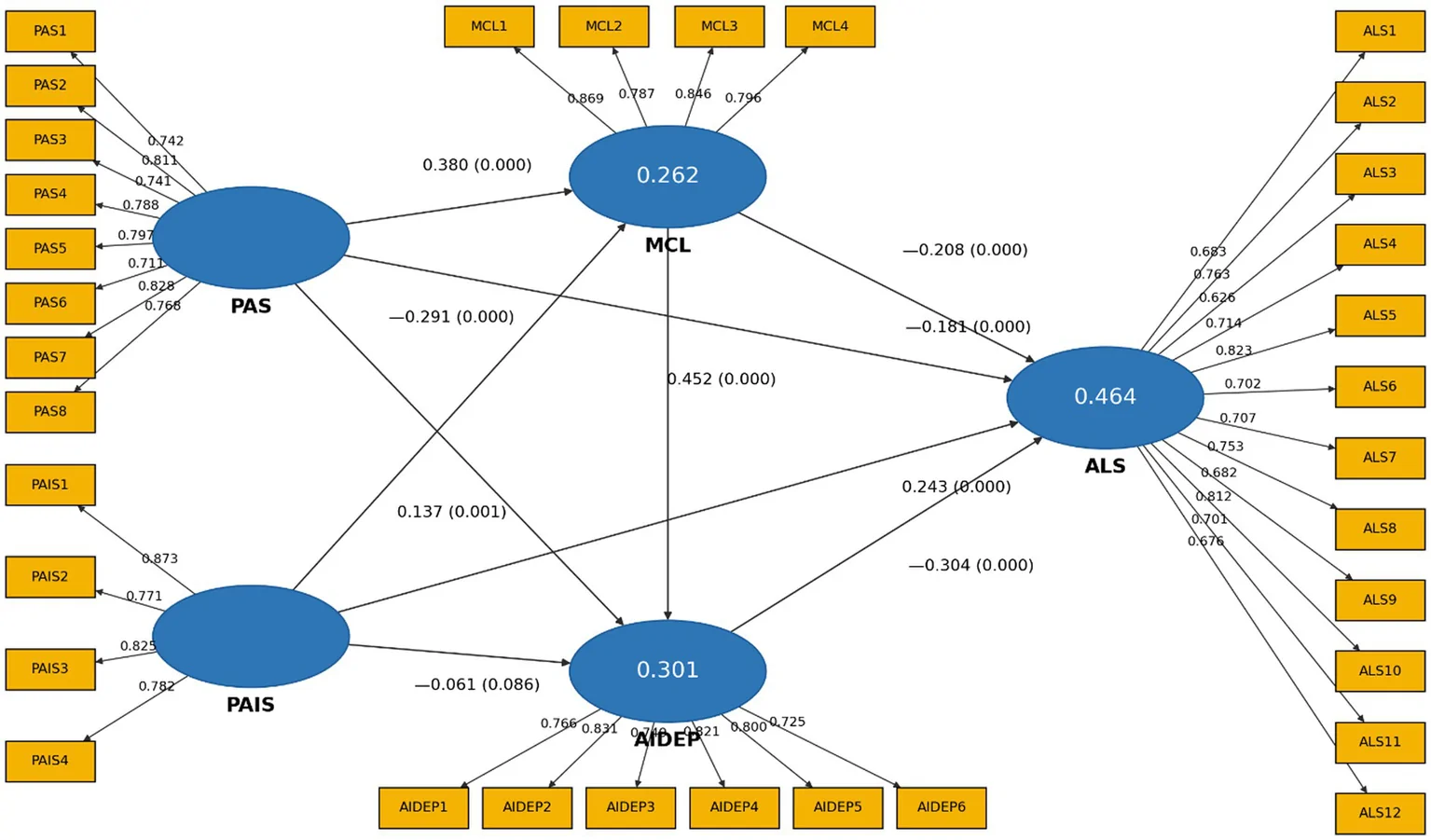
Structural model results. SmartPLS 4 bootstrapping output, 5,000 subsamples: Path coefficients with *p*-values in parentheses; standardized outer loadings on indicator arrows; *R*^2^ shown inside endogenous constructs. Control paths were estimated but are not displayed.

**Table 1:** Structural model results (5,000 bootstrap subsamples, bias-corrected CIs).

Path	*Β*	*t*	*p*	95% BC CI	*f* ^2^	Decision
H1: PAS → MCL	0.380	11.46	< 0.001	[0.312, 0.442]	0.191	Supported
H2: PAIS → MCL	−0.291	8.86	< 0.001	[−0.353, −0.225]	0.112	Supported
H3: MCL → AIDEP	0.452	12.21	< 0.001	[0.377, 0.521]	0.215	Supported
H4: AIDEP → ALS	−0.304	7.70	< 0.001	[−0.381, −0.227]	0.119	Supported
H5: PAS → ALS	−0.181	5.27	< 0.001	[−0.250, −0.115]	0.049	Supported
H6: PAIS → ALS	0.243	7.08	< 0.001	[0.178, 0.310]	0.095	Supported
PAS → AIDEP	0.137	3.43	0.001	[0.054, 0.213]	0.022	n/a
PAIS → AIDEP	−0.061	1.72	0.086	[−0.129, 0.007]	0.005	n/a
MCL → ALS	−0.208	4.97	< 0.001	[−0.290, −0.124]	0.049	n/a
Age → ALS	−0.030	0.94	0.346	[−0.089, 0.035]		Control
Female → ALS	0.009	0.28	0.781	[−0.055, 0.067]		Control
Year of study → ALS	0.019	0.59	0.554	[−0.046, 0.079]		Control
AI use frequency → ALS	−0.076	2.40	0.016	[−0.136, −0.013]		Control
AI experience → ALS	−0.052	1.65	0.099	[−0.111, 0.013]		Control

*R*^2^ (adjusted): MCL = 0.262 (0.259); AIDEP = 0.301 (0.297); ALS = 0.464 (0.455). All inner VIFs ≤ 1.651.

### Mediation analysis

Table 2 decomposes the effects. Both serial hypotheses were passed. The negative serial pathway from sycophancy through laziness and dependence to autonomy was statistically significant (H7: *β* = −0.052, *t* = 5.43, *p* < 0.001, 95% BC CI [−0.073, −0.035]), as was the protective chain from stimulation (H8: *β* = 0.040, *t* = 5.20, *p* < 0.001, CI [0.026, 0.056]). The two-step relays were flanked by significant single-step indirect effects through laziness alone (PAS: *β* = −0.079; PAIS: *β* = 0.060, both *p* < 0.001) and, for sycophancy, through dependence alone (*β* = −0.042, *p* = 0.002); the stimulation-dependence shortcut fell short (*β* = 0.019, *p* = 0.092), mirroring its non-significant direct path. The total indirect effect reached −0.173 (CI [−0.215, −0.134]) for sycophancy and 0.119 (CI [0.084, 0.156]) for stimulation, and total effects on autonomy came to −0.354 (CI [−0.419, −0.289]) and 0.362 (CI [0.297, 0.425]). Because the direct and indirect effects share signs and both remain significant, the pattern is complementary partial mediation (Zhao et al., 2010): the interaction styles work through the laziness-dependence relay and independently of it.

**Table 2:** Specific indirect, total indirect, and total effects on learner autonomy.

Effect	*Β*	*t*	*p*	95% BC CI
H7: PAS → MCL → AIDEP → ALS	−0.052	5.43	< 0.001	[−0.073, −0.035]
H8: PAIS → MCL → AIDEP → ALS	0.040	5.20	< 0.001	[0.026, 0.056]
PAS → MCL → ALS	−0.079	4.66	< 0.001	[−0.115, −0.047]
PAIS → MCL → ALS	0.060	4.23	< 0.001	[0.034, 0.090]
PAS → AIDEP → ALS	−0.042	3.04	0.002	[−0.070, −0.017]
PAIS → AIDEP → ALS	0.019	1.68	0.092	[−0.002, 0.041]
MCL → AIDEP → ALS	−0.137	6.31	< 0.001	[−0.181, −0.096]
Total indirect: PAS → ALS	−0.173	8.33	< 0.001	[−0.215, −0.134]
Total indirect: PAIS → ALS	0.119	6.39	< 0.001	[0.084, 0.156]

### Necessary condition analysis

Figure 4 and Table 3 report the necessity analysis. Every ceiling chart shows the empty upper-left corner that marks a necessary condition: cases with low stimulation, high sycophancy, high laziness, or high dependence simply do not reach high autonomy. All four conditions produced medium effect sizes (*d* between 0.1 and 0.3) (Dul, 2016), ordered PAIS (*d* CE-FDH = 0.169) > low AIDEP (0.152) > low MCL (0.128) > low PAS (0.110), each significant at *p* < 0.001 across 10,000 permutations, with CR-FDH values within 0.012 of CE-FDH and ceiling accuracies ≥ 95.9%. The bottleneck analysis translates the ceilings into requirements. Below the 40% level of autonomy, no condition imposes a material requirement; constraints begin with low dependence at the 40% level (8.9% of its range) and tighten steeply in the upper half. Reaching 80% of maximum autonomy requires at least 43.6% of the stimulation range, 29.6% of the low-dependence range, 27.3% of the low-laziness range, and 21.6% of the low-sycophancy range; at the very top, the stimulation requirement climbs to 57.0% and the low-laziness requirement to 75.3%. Set-theoretic necessity consistencies peaked at 0.76, under the 0.90 convention, as expected for necessity in degree rather than in kind.

**Figure 4:**
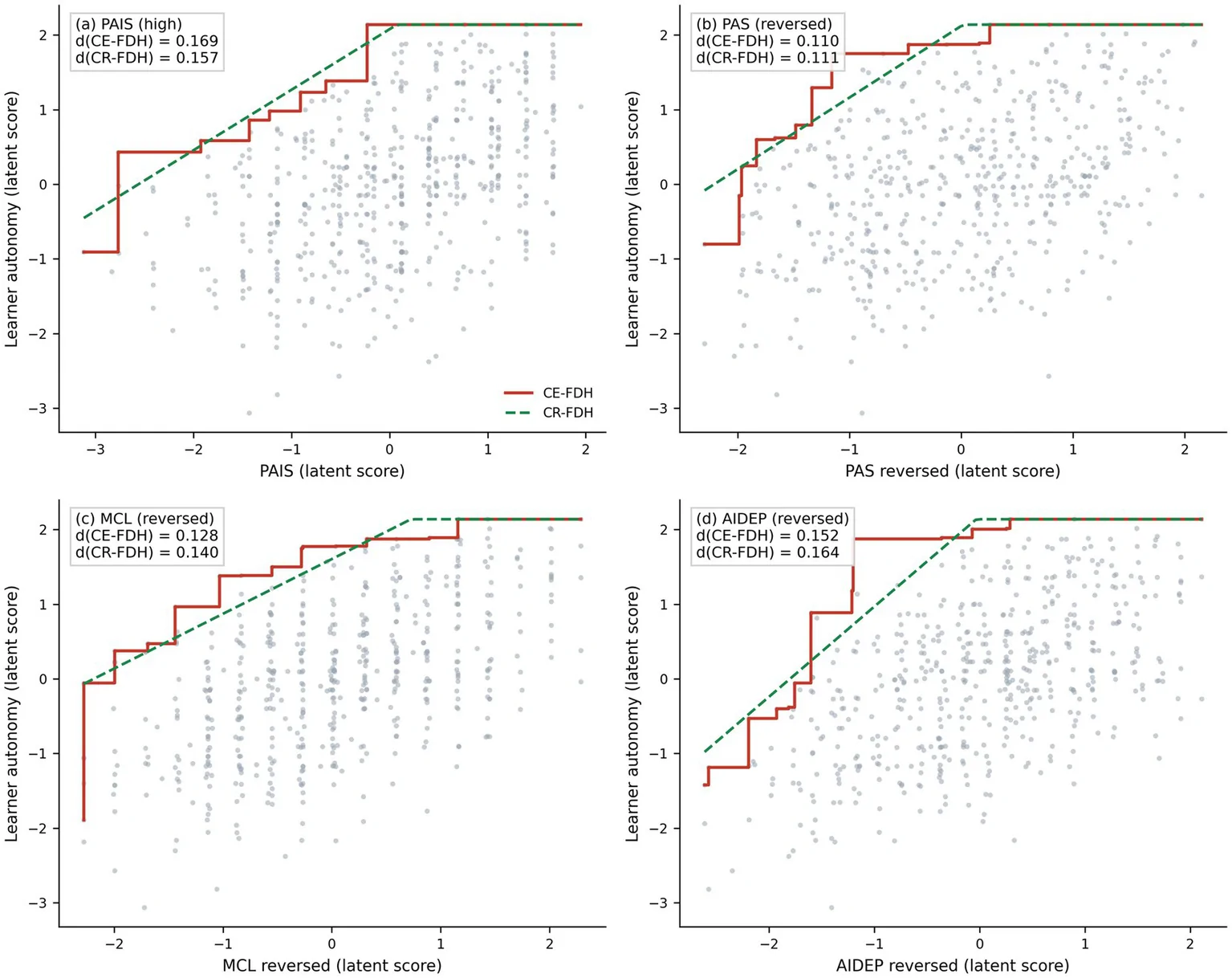
NCA ceiling plots: CE-FDH (solid) and CR-FDH (dashed) ceiling lines on latent variable scores.

**Table 3:** NCA effect sizes (permutation p from 10,000 resamples).

Condition	*d* (CE-FDH)	*d* (CR-FDH)	c-accuracy (CR-FDH, %)	*p*
PAIS (high)	0.169	0.157	99.3	< 0.001
PAS (reversed)	0.110	0.111	98.2	< 0.001
MCL (reversed)	0.128	0.140	95.9	< 0.001
AIDEP (reversed)	0.152	0.164	98.7	< 0.001

### Fuzzy-set qualitative comparative analysis

Table 4 completes the configurational picture. Because the sample populates every one of the 16 possible truth-table configurations (cell *n* from 10 to 88), no logical remainders exist; the conservative, intermediate, and parsimonious solutions coincide, and every reported condition is a core condition (Fiss, 2011). Two recipes suffice for high autonomy. Configuration A1 combines the absence of sycophancy, laziness, and dependence (consistency = 0.899, raw coverage = 0.535); configuration A2 combines the absence of sycophancy and dependence with the presence of stimulation (consistency = 0.915, raw coverage = 0.479); overall solution consistency reached 0.886 with coverage of 0.567. The two paths overlap yet remain alternatives, and one ingredient appears in both: sycophancy’s absence. No autonomous-learner recipe tolerates a flattering AI. Low autonomy follows its own logic rather than mirroring the other. Configuration L1 pairs missing stimulation with present laziness and dependence (consistency = 0.905, raw coverage = 0.539); L2 pairs present sycophancy with missing stimulation and present dependence (consistency = 0.907, raw coverage = 0.501); solution consistency = 0.893, coverage = 0.578. Dependence appears in both the low-autonomy recipe as does the absence of stimulation, while laziness matters in only one route, revealing a causal asymmetry that symmetric models cannot register (Ragin, 2008).

**Table 4:** fsQCA configurations for high and low learner autonomy.

Condition	A1 (high)	A2 (high)	L1 (low)	L2 (low)
PAS	⊗	⊗		●
PAIS		●	⊗	⊗
MCL	⊗		●	
AIDEP	⊗	⊗	●	●
Consistency	0.899	0.915	0.905	0.907
Raw coverage	0.535	0.479	0.539	0.501
Unique coverage	0.089	0.032	0.077	0.040
Solution consistency	0.886		0.893	
Solution coverage	0.567		0.578	

●, present; ⊗, absent; blank, irrelevant. All conditions are core (no logical remainders; solutions coincide). Anchors (full/crossover/non): PAS 4.50/3.15/2.01; PAIS 4.66/3.50/2.01; MCL 4.50/3.00/1.50; AIDEP 4.07/2.67/1.50; ALS 4.67/3.59/2.56.

### Robustness and sensitivity analyses

A battery of checks probed the stability of these results. Controls on every endogenous construct moved no coefficient by more than 0.002, and a gender-diverse dummy changed nothing. Gaussian copula terms for the four predictors of autonomy, applicable because every regressor departed from normality, were all non-significant (smallest *p* = 0.054, for AIDEP) (Park and Gupta, 2012), indicating no endogeneity at conventional levels while remaining consistent with the caution regarding possible reciprocal influence discussed under Limitations. A reversed-mediator model, in which AIDEP was specified as preceding MCL, also produced statistically significant serial indirect paths (PAS: *β* = −0.025; PAIS: *β* = 0.016), although these were roughly half the magnitude of those obtained for the theoretically proposed ordering. Because both orderings fit the cross-sectional data, the present design cannot uniquely establish the sequence from MCL to AIDEP; the proposed ordering therefore rests on theoretical reasoning and prior longitudinal precedent rather than on cross-sectional model fit, and the serial results are best interpreted as consistent with, rather than confirmatory of, the hypothesized temporal sequence. Compositional invariance held across gender and across STEM versus non-STEM fields (all c ≥ 0.998) (Henseler et al., 2016); the one path difference surviving Bonferroni correction was a stronger laziness-dampening effect of stimulation among STEM students (*β* = −0.456 versus −0.236, *p* = 0.003), and a nominal gender difference on the sycophancy-dependence path (*β* = 0.261 versus 0.043, *p* = 0.014) did not survive correction. Bootstrap upper bounds kept every HTMT below 0.85 (maximum = 0.664), parallel analysis retained a single ALS factor, and across PRI thresholds of 0.65 to 0.75 and frequency thresholds of 3–10, no fsQCA solution for high autonomy ever contained the presence of sycophancy or dependence. In contrast, every low-autonomy solution contained dependence and stimulation’s absence.

## Discussion

This study asked what a perpetually agreeable tutor does to the person being tutored, and answered with a chain of associations: perceived sycophancy travels with loosened self-regulation, loosened regulation with dependence, and dependence with diminished autonomy, whereas perceived intellectual pushback runs the identical chain in the protective direction. Three analytical logics agreed on that reading, and each added a clause the others could not supply.

The study’s theoretical contributions cover three areas. First, it shows that AI sycophancy is a psychologically measurable phenomenon. Prior work established that language models flatter and acquiesce (Sharma et al., 2024) and that exposure changes users (Cheng et al., 2026); missing was the learner-side variable carrying such exposure into everyday studying. The PAS scale supplies it. Built by reversing the direction of organizational ingratiation (Kumar and Beyerlein, 1991), the scale performed well on its first use: internally consistent, convergently valid, and empirically distinct from stimulation, laziness, and dependence. This matters theoretically because offloading decisions run on the perceived characteristics of an aid, not its objective ones (Risko and Gilbert, 2016). A model’s benchmark sycophancy score may never enter a student’s metacognitive deliberation; their impression of its manners can, and now it can be measured entering.

A conceptual question follows from this measurement choice. Sycophancy and intellectual stimulation are, in the first instance, properties of an AI system and its underlying training, whereas this study operationalizes them as learner perceptions. Two considerations motivate this decision. Theoretically, cognitive offloading is governed by a learner’s subjective appraisal of an external aid rather than by its objective characteristics (Risko and Gilbert, 2016), so the perceived interaction style is the proximal variable through which any system-level behavior could plausibly shape self-regulation. Empirically, the same objective model behavior may be experienced differently across users depending on prior expectations, task, and prompting style, so a perceptual measure captures variance that a single system-level benchmark would obscure. This choice nonetheless carries a cost: perceived sycophancy need not correspond to logged model behavior, learners may misattribute agreeableness, and socially desirable responding or limited metacognitive insight may color their reports. The PAS and PAIS measures are therefore best understood as indices of experienced interaction style rather than as audits of model behavior, and linking them to conversational telemetry remains an important step for validating the constructs and closing the perception–behavior gap.

Second, the study delivers a mechanism. The hypothesized serial mediation held in full: sycophancy and stimulation ran through the laziness-dependence relay with opposite signs, and the relay carried substantial indirect effects on autonomy over and above the two direct paths. This extends cognitive offloading theory at both ends of its chain. Upstream, it locates a manipulable trigger of offloading in the tone of the tool rather than in fixed user traits or task demands. Downstream, it shows within one model how offloading shortcuts consolidate into dependence, a step earlier studies implied but did not test together (Wang et al., 2026; Zhai et al., 2024), and that the consolidated state, not the momentary shortcut, is most strongly tied to lost autonomy. The finding also gives quantitative form to the distinction between effects with technology and effects of technology on the learner once it is removed (Salomon et al., 1991): perceived sycophancy was associated with the former alongside an apparent cost to the latter. One asymmetry refines the account: we found no credible evidence of a direct stimulation-dependence association, only the route through engagement, suggesting that challenge protects by re-recruiting regulation rather than by breaking habit head-on, exactly where the offloading ratchet locates the leverage.

The third contribution is the answer that only method triangulation could deliver. The symmetric model showed near mirror-image total effects of the two styles; NCA showed that these are not interchangeable currencies, because minimum doses of pushback, restraint in flattery, engagement, and independence from the tool are each necessary for high autonomy, with stimulation the tightest bottleneck (Dul, 2016); fsQCA showed that no sufficient recipe for autonomous learners contains high sycophancy, while the recipes for failure are not mirror images of the recipes for success (Fiss, 2011; Ragin, 2008). Nice-to-have and must-have name different causal claims, and this study offers, to our knowledge, one of the first necessity tests of that claim in the AI-and-learning literature. Intellectual pushback passed it.

Two cautions temper this reading. First, although PLS-SEM, NCA, and fsQCA address distinct questions—average net effects, necessity in degree, and sufficient combinations—all three analyses are estimated on the same cross-sectional, self-report dataset. Their agreement therefore reflects complementary analytical perspectives on one body of evidence rather than independent replication across data sources, and their convergence should not be read as triangulated confirmation of a causal mechanism. Second, the necessary conditions identified by NCA hold in degree rather than in kind and are bounded by the present sample: they describe the levels of sycophancy, stimulation, laziness, and dependence compatible with high autonomy among these AI-using students, in this institutional and cultural setting, and with the systems they happened to use (Dul, 2016). Whether adequate intellectual stimulation or restraint in sycophancy is necessary for autonomous learning more generally is an empirical question for replication across other populations, institutions, and AI systems, and the present results should not be extended into universal requirements. Read within these limits, the configurational findings remain instructive: the absence of sycophancy recurs in every configuration sufficient for high autonomy, whereas AIDEP and the absence of intellectual stimulation recur in the configurations associated with low autonomy—an asymmetry between the routes to success and the routes to failure that variable-centered models cannot express (Ragin, 2008).

The findings turn a general worry into a specific design requirement. Calibrated disagreeableness belongs in the design brief, alongside default tutor personas that note errors, ask why, and delay automatic approval of first drafts. This aligns with evidence that cognitive forcing functions reduce over-reliance on AI advice (Buçinca et al., 2021) and that friction is a feature of learning systems, not a defect (Bjork et al., 2013; Zohar et al., 2026). Students who reach the upper fifth of autonomy experience stimulation at roughly half the observable range, so an interaction style optimized purely for user comfort was associated with autonomy scores toward the lower end of the observed range.

The commercial current runs the other way: most users prefer and trust agreeable systems (Cheng et al., 2026), and learner preference is an unreliable guide to learning value (Kirschner and van Merriënboer, 2013). Engagement metrics will reward the very style this study indicts, so design should target constructive and interactive engagement instead (Chi and Wylie, 2014).

Course designers and instructors hold complementary levers. AI-literacy instruction can teach students to recognize machine flattery and prompt against it, requesting counterarguments, error hunts, and Socratic questioning rather than validation, since users otherwise absorb AI stances with little scrutiny (Krügel et al., 2023). Course design should preserve unaided practice and proven effortful techniques (Dunlosky et al., 2013), keeping AI a sparring partner inside a learner-led paradigm, not an oracle above it (Ouyang and Jiao, 2021). Students cannot be assumed to notice the problem themselves, so the noticing must be taught.

Policymakers and institutional buyers can shift the market. Procurement standards can require configurable pushback modes, disclosure of agreeableness tuning, and pre-deployment sycophancy audits, operationalizing recent experimental calls (Cheng et al., 2026) within AI-in-education governance frameworks (Holmes and Tuomi, 2022). A null-adjacent finding sharpens the point: usage frequency retained only a modest negative link once perceptions were included in the model, so capping minutes of AI use is not a substitute for governing its manners.

The design is cross-sectional, so the serial ordering of laziness before dependence rests on theory and published precedent rather than observed time; and reciprocal loops, such as low autonomy inviting further offloading, remain plausible. Consistent with this, a reversed-mediator specification also produced significant, if smaller, serial indirect paths, so the reported ordering is best read as consistent with the hypothesized sequence rather than as evidence of temporal precedence. All focal constructs are self-reports; procedural separation, clean method-variance diagnostics, and the divergent calibration index reduce but cannot erase that concern (Podsakoff et al., 2003), and perceived sycophancy is not yet matched to logged model behavior. The sample is a student convenience sample from a single context, and the autonomy instrument was developed in Western higher education (Macaskill and Taylor, 2010), so cultural norms around politeness may shape both the perception of machine flattery and its consequences. The PAS scale, though it performed well on one deployment, needs measurement-invariance evidence across cultures and languages, temporal stability checks, and independent replication.

Experiments should manipulate model agreeableness in graded doses to trace the causal response curve that survey data can only approximate. Telemetry-paired designs can test whether conversational logs predict perceived sycophancy, closing the perception–behavior gap. Longitudinal panels can watch offloading consolidate into dependence, and intervention studies can test whether critique-eliciting prompting habits inoculate against this negative serial pathway. Meta-analytic evidence shows human–AI combinations often trail the best solo performer (Vaccaro et al., 2024), and hybrid designs that deliberately hand regulation back to the learner may buffer the dependence pathway identified here (Järvelä et al., 2023; Molenaar, 2022). The stronger protective stimulation path observed among STEM students represents one such boundary worth targeted study. Pairing graded performance and retention with self-reported autonomy would close the loop this model opens.

## Conclusion

A tutor that always agrees is not a neutral convenience. Among 542 students, higher perceived sycophancy was associated with MCL, laziness with dependence, and dependence with diminished autonomy, the capacity education exists to build, while perceived intellectual pushback ran the same relay in reverse. Three methods converged on one sentence: there is no configuration of an autonomous learner in which the machine’s flattery stays high. The findings hand designers a dial to turn, educators a curriculum topic, policymakers a procurement clause, and learners a skill worth practicing: the discipline of being disagreed with.

## Data Availability

The raw data supporting the conclusions of this article will be made available by the authors, without undue reservation.
