# Supplementary material for: How AI sycophancy shapes learner autonomy in digitalized learning: the mediating roles of metacognitive laziness and AI dependence
Source: Front Psychol. 2026 Sep 30;17:1957447. doi: 10.3389/fpsyg.2026.1957447 (PMC13662133; doi:10.3389/fpsyg.2026.1957447)
Supplement: Supplementary file 1 [file Supplementary_file_1.docx]

**Supplementary Information**

***Supplementary Note S1. Full item wording***

All focal constructs were measured on five-point Likert-type scales. Response options ranged from 1 (strongly disagree) to 5 (strongly agree), except for the perceived AI intellectual stimulation items, which used frequency anchors from 1 (never) to 5 (always). Items are listed below by construct. Items were coded so that higher scores indicate more of each construct, and no reverse-keyed items were retained in the administered form.

**Perceived AI Sycophancy (PAS) – 8 items (newly adapted from Kumar & Beyerlein, 1991)**
1. The AI agrees with my opinions and judgments, even when I might be wrong.
2. The AI compliments my ideas regardless of their actual quality.
3. The AI rarely tells me directly that I am mistaken.
4. When I disagree, the AI quickly abandons its original answer to match my view.
5. The AI tends to tell me what I want to hear rather than what is accurate.
6. The AI praises my work even when it contains clear errors.
7. The AI softens or withdraws its criticism as soon as I push back.
8. The AI reassures me that my reasoning is sound without examining it closely.

**Perceived AI Intellectual Stimulation (PAIS) – 4 items (adapted from the Transformational Teaching Questionnaire, Beauchamp et al., 2010)**
Stem: “My AI tutor …”
1. gives responses that really encourage me to think.
2. challenges me to reconsider assumptions I had not questioned.
3. prompts me to look at problems from new perspectives.
4. encourages me to work through problems on my own rather than simply giving answers.

**Metacognitive Laziness (MCL) – 4 items (adapted from the Metacognitive Laziness Scale, Dizon et al., 2026)**
1. I avoid challenging learning tasks when AI can do them for me.
2. When I use AI, I put little effort into planning how to approach my work.
3. I rely on AI to check whether my work is correct instead of checking it myself.
4. I let AI judge the quality of my work rather than evaluating it myself.

**AI Dependence (AIDEP) – 6 items (adapted from Zhang et al., 2024)**
1. I tried to reduce the use of ChatGPT without success.
2. I feel uneasy when I cannot use AI tools for my studies.
3. I use AI tools for longer than I originally intend.
4. I find it difficult to complete academic tasks without AI assistance.
5. My use of AI tools interferes with other things I should be doing.
6. I keep using AI tools for coursework even when I think I should rely on myself.

**Learner Autonomy (ALS) – representative items (full 12-item Autonomous Learning Scale, Macaskill & Taylor, 2010)**
1. I enjoy seeking out information on new topics on my own.
2. I plan and manage my study time effectively.
3. I take responsibility for my own learning.
4. I keep working at a task until I am satisfied that it is done well.

Note. Learner autonomy was measured with the complete 12-item Autonomous Learning Scale (Macaskill & Taylor, 2010) administered with its original item wording; four representative items are shown here, and the full instrument is available in the original source. Full item wording for the adapted PAS, PAIS, MCL, and AIDEP scales is provided above.

***Supplementary Note S2. Distribution of respondents across participating universities***

Table S1 reports the number and percentage of respondents recruited from each of the ten participating universities. No single institution contributed a dominant share of the sample, and the structural results were substantively unchanged when institution was entered as a control variable (|Δβ| ≤ 0.002, all p > .05).

**Table S1.** *Institutional distribution of the study sample (N = 542).*

| **University** | **Frequency (n)** | **Percentage (%)** |
| --- | --- | --- |
| University of Ha'il | 84 | 15.5% |
| King Saud University | 76 | 14.0% |
| King Abdulaziz University | 71 | 13.1% |
| King Fahd University of Petroleum and Minerals (KFUPM) | 58 | 10.7% |
| Umm Al-Qura University | 53 | 9.8% |
| Imam Mohammad Ibn Saud Islamic University | 49 | 9.0% |
| Princess Nourah bint Abdulrahman University | 44 | 8.1% |
| Taibah University | 39 | 7.2% |
| King Khalid University | 36 | 6.6% |
| Jouf University | 32 | 5.9% |
| **Total** | **542** | **100.0%** |
